# Alternative signal pathways underly fertilization and egg activation in a fish with contrasting modes of spawning

**DOI:** 10.1186/s12864-023-09244-1

**Published:** 2023-04-04

**Authors:** Feng Chen, Yeke Wang, Jun He, Carl Smith, Ge Xue, Yan Zhao, Yanghui Peng, Jia Zhang, Jiarui Liu, Jun Chen, Ping Xie

**Affiliations:** 1 State Key Laboratory of Freshwater Ecology and Biotechnology, Institute of Hydrobiology, Donghu Experimental Station of Lake Ecosystems, Chinese Academy of Sciences, 430072 Wuhan, China; 2 Life Sciences Institute, Zhejiang University, 310058 Hangzhou, China; 3 Department of Ecology and Vertebrate Zoology, University of Łódź, Łódź, Poland; 4 Institute of Vertebrate Biology, Academy of Sciences of the Czech Republic, Brno, Czech Republic; 5 University of Chinese Academy of Sciences, 100049 Beijing, China; 6 Institute of Ecological Research and Pollution Control of Plateau Lakes, School of Ecology and Environment, Yunnan University, 650500 Kunming, China

**Keywords:** Fertilization, Egg activation, Signal pathway, Spawning mode, Teleost

## Abstract

**Background:**

The processes of fertilization and egg activation are vital for early embryogenesis. However, while the mechanisms associated with key events during these processes differ among species and modes of spawning, the signal pathways underlying these processes are opaque for many fishes, including economically important species.

**Results:**

We investigated phenotypic traits, ultrastructure and protein expression levels in the eggs of the topmouth culter (*Culter alburnus*), a protected and economically important freshwater fish that exhibits two spawning modes, producing semi-buoyant eggs and adhesive eggs. Unfertilized eggs of *C. alburnus* were examined, as well as eggs at fertilization and 30 min post fertilization. Our results showed that in semi-buoyant eggs, energy metabolism was activated at fertilization, followed by elevated protein expression of cytoskeleton and extracellular matrix (ECM)-receptor interactions that resulted in rapid egg swelling; a recognized adaptation for lotic habitats. In contrast, in adhesive eggs fertilization initiated the process of sperm-egg fusion and blocking of polyspermy, followed by enhanced protein expression of lipid metabolism and the formation of egg envelope adhesion and hardening, which are adaptive in lentic habitats.

**Conclusion:**

Our findings indicate that alternative signal pathways differ between modes of spawning and timing during the key processes of fertilization and egg activation, providing new insights into the molecular mechanisms involved in adaptive early embryonic development in teleost fishes.

**Supplementary Information:**

The online version contains supplementary material available at 10.1186/s12864-023-09244-1.

## Background

The processes of fertilization and egg activation are fundamental developmental events in the early embryogenesis of animals. The basic morphological and physio-biochemical traits associated with fertilization and egg activation are highly conserved, and involve principles that are common to most vertebrates, including fishes [1–5]. During early embryonic development of fishes, the physiological events of intracellular Ca^2+^ oscillations, sperm-egg fusion, cortical reaction, polyspermy blocks, and egg swelling, hardening and adhesion contribute to fertilization and egg activation [3, 6–10]. Although much of our understanding of fish egg morphology and physio-biochemistry comes from commercially-important species, our recognition of the molecular mechanisms governing fertilization and egg activation is largely confined to small model species, such as zebrafish (*Danio rerio*), medaka (*Oryzias latipes*) and rose bitterling (*Rhodeus ocellatus*).

At fertilization, egg activation is usually triggered by an increase in the intracellular Ca^2+^ concentration in the egg [1, 3, 7]. Three models of the molecular mechanisms underlying how the sperm induces intracellular Ca^2+^ release within the egg are proposed, and include the ‘Ca^2+^ bomb’ model, ‘membrane receptor’ model, and ‘soluble sperm factor’ model [7, 11–13]. The breakdown of cortical alveoli is also associated with a large increase in intracellular Ca^2+^ ions [14]. It is suggested that the contents of these alveoli, including glycosaminoglycans [15], polysaccharides [16], endogenously derived glycoprotein [17], lipoprotein [18] and lectins with sugar binding properties [19], function to prevent polyspermy, harden or elevate the egg envelope and produce adhesiveness [9, 20, 21]. However, the mechanisms associated with these events during fertilization vary among species. For example, egg activation in zebrafish is initiated only through contact with the external spawning medium [22], while medaka eggs appear only to require direct contact with conspecific sperm [23]. Given our limited understanding of the adaptive basis to this variation in the molecular mechanisms underpinning egg activation and fertilization, a better understanding of these mechanisms and their adaptive and evolutionary context is essential.

External fertilization of fish eggs occurs in an extraordinarily wide variety of environmental settings, with the mechanisms of egg activation and fertilization during early embryogenesis also showing variation [3, 7]. Notably, the eggs of fish with different spawning modes show a significant variety of adaptations to the environments in which they are found. The fertilization of semi-buoyant eggs always occurs in fast-flowing rivers during the flood season, while adhesive eggs are fertilized on aquatic vegetation, gravel or other hard surfaces in lentic habitats [9, 24]. During early embryogenesis, semi-buoyant eggs rapidly take up water to form a large perivitelline space, which has the function of lifting the egg into suspension in the water column during development. In contrast, adhesive eggs produce an adhesive layer and adhere to aquatic plants or other hard surfaces [25, 26]. Despite the clear functional differences in semi-buoyant and adhesive eggs, the different molecular pathways underlying fertilization and egg activation between these spawning modes have yet to be identified. Part of the problem in identifying such differences is that, while there is wide variation among species, within-species variation is typically absent, which limits the ability to untangle differences in molecular pathways between spawning modes from broader species differences [27, 28]. However, some species exhibit alternative spawning modes within species and offer tractable material for isolating the molecular pathways that underpin fertilization and egg activation in different spawning modes.

The topmouth culter (*Culter alburnus* Basilewsky, 1855) (Cypriniformes: Cyprinidae: Cultrinae), is a freshwater teleost fish with two spawning modes. It is a widespread and economically important species in China and has received national key protection since 2007. Some fish produce semi-buoyant eggs in lotic environments, while others spawn adhesive eggs in lentic environments [29], thereby making *C. alburnus* an ideal model to examine differences in the signal pathways involved in fertilization and egg activation between semi-buoyant and adhesive eggs.

In this study, we examined phenotypic traits, ultrastructure and protein levels in semi-buoyant and adhesive eggs of *C. alburnus* at three stages; unfertilized (Un-FE), immediately following fertilization (0 min post fertilization (0 min-FE)), and 30 min post fertilization (30 min-FE). As the key events accompanying fertilization and egg activation occur during early embryogenesis, before the mid-blastula transition, gene expression of the egg originates predominantly from maternal RNA, with a limited contribution from zygotic transcripts [30–32]. Consequently, we used tandem mass tag (TMT)-based protein quantification techniques, which are appropriate for providing precise information on early embryogenesis. We aimed to identify changes in the morphological characteristics and signal pathways between fertilization and egg activation stages, and reveal differences in enriched signal pathways associated with different spawning modes in *C. alburnus*. These findings potentially offer new insights into the molecular mechanisms involved in adaptive early embryonic development of teleost fishes.

## Results

### Phenotypic traits

**Fig. 1:**
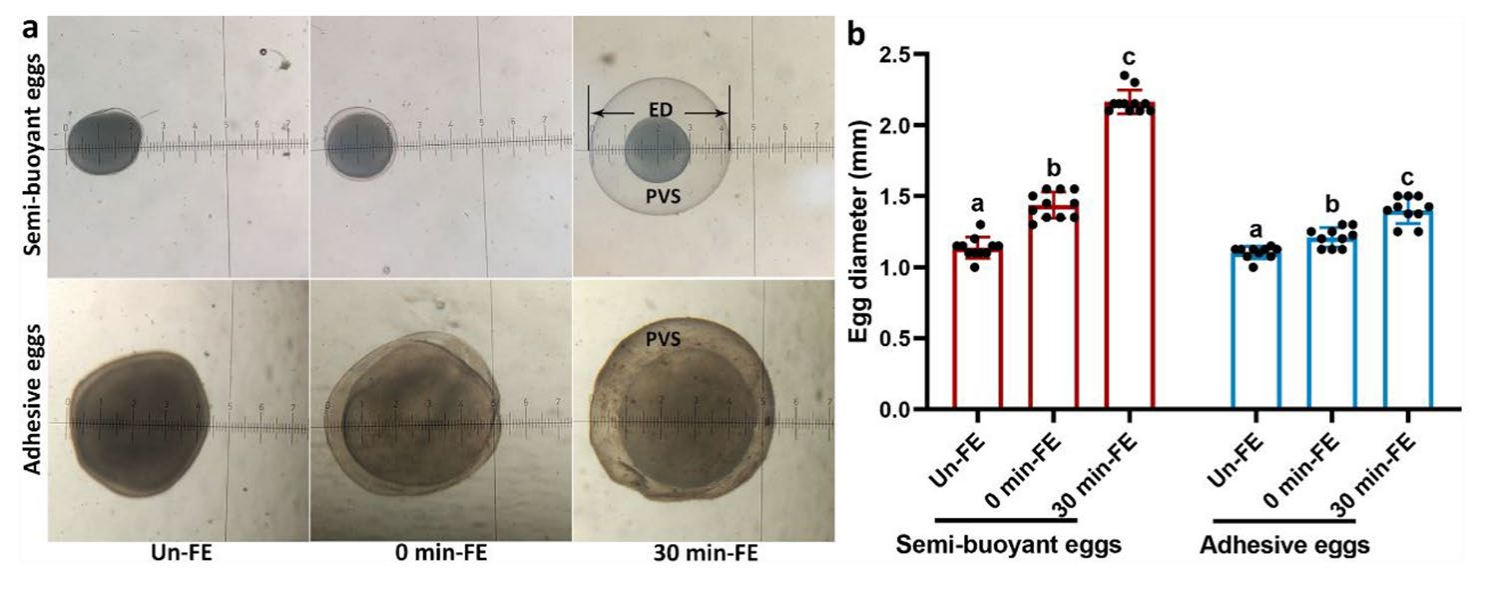
The phenotypic traits of semi-buoyant and adhesive eggs of *C. alburnus* at three time stages of unfertilized (Un-FE), and 0 and 30 min post fertilization (0 min-FE and 30 min-FE). a, phenotypic appearance of semi-buoyant (original magnification × 20) and adhesive eggs (original magnification × 40) viewed under a stereomicroscope. ED, egg diameter; PVS, perivitelline space. b, egg diameters of semi-buoyant and adhesive eggs. Values are mean ± SD (n = 10 or 11). Different lower-case letters indicate significant differences between time stages (*p* < 0.05, one-way analysis of variance)

Under a stereomicroscope, semi-buoyant eggs of *C. alburnus* were observed to rapidly take up water following fertilization and remained transparent, while adhesive eggs also hydrated but were opaque after fertilization (Fig. 1a). The egg diameters of semi-buoyant and adhesive eggs increased significantly following fertilization (Fig. 1b).

### Ultrastructure of eggs

**Fig. 2:**
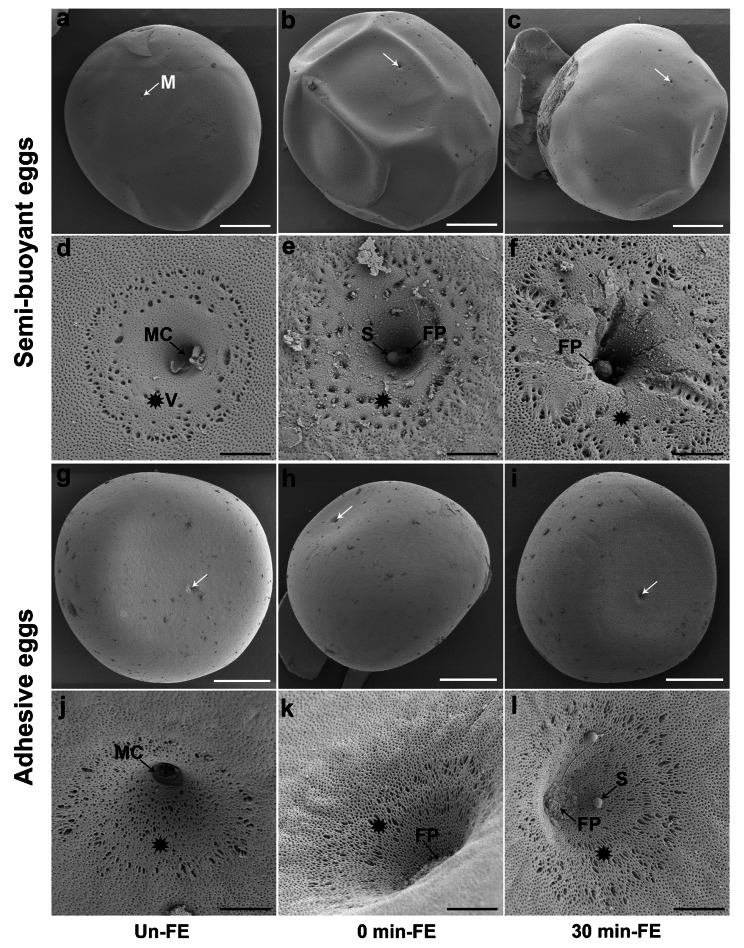
SEM images of semi-buoyant and adhesive eggs in *C. alburnus* at three time stages of unfertilized (Un-FE), and 0, and 30 min post fertilization (0 min-FE and 30 min-FE). a-f show images of semi-buoyant eggs (white scale bar = 250 μm, black scale bar = 10 μm); g-l show images of adhesive eggs (white scale bar = 250 μm, black scale bar = 10 μm). White arrows indicate micropyle (M). Black stars indicate vestibule (V). MC, micropylar canal; S, sperm; FP, fertilization plug

Scanning electron microscopy (SEM) images showed that semi-buoyant and adhesive eggs of *C. alburnus* typically possessed a single micropyle, which was funnel-shaped (Fig. 2). The micropylar canal was open in the unfertilized egg (Fig. 2d&j), and closed by a fertilization plug following fertilization (Fig. 2f). The edge of the vestibule was surrounded by significantly enlarged pore openings (Fig. 2d-f&j-l). Large pore openings were more dense at the interior of the vestibule in adhesive eggs, but were more sparsely distributed in semi-buoyant eggs. The vestibule was flattened and narrower in semi-buoyant eggs, while it was recessed and wider in adhesive eggs.

### Quantification of egg proteins

A total of 5,754 proteins were quantified among the total of 7,017 identified egg proteins of *C. alburnus*. Among the quantified differentially expressed proteins (a fold change > 1.2 or < 0.83 and *p* < 0.05), there were 143 up-regulated proteins (URPs) and 89 down-regulated proteins (DRPs) in unfertilized eggs compared with eggs at 0 min-FE, and 61 URPs and 342 DRPs in eggs at 0 min-FE relative to 30 min-FE in semi-buoyant eggs (PE) (Table 1; see Additional file 1). In adhesive eggs (AE), 175 URPs and 103 DRPs were identified in unfertilized eggs compared to eggs at 0 min-FE, and 90 URPs and 68 DRPs in eggs at 0 min-FE in comparison with those at 30 min-FE (Table 1; see Additional file 1). DRPs in semi-buoyant and adhesive eggs of *C. alburnus* for each of these pair-wise comparisons were investigated in this study.

**Table 1:** The number of differentially expressed proteins between unfertilized (Un-FE), and 0 and 30 min post fertilization (0 min-FE and 30 min-FE) in semi-buoyant eggs (PE) and adhesive eggs (AE) of *C. alburnus*

Groups compared	Up-regulated	Down-regulated
PE-Un-FE vs. PE-0 min-FE	134	89
PE-0 min-FE vs. PE-30 min-FE	61	342
AE-Un-FE vs. AE-0 min-FE	175	103
AE-0 min-FE vs. AE-30 min-FE	90	68

### Functional classification of differentially expressed proteins

**Fig. 3:**
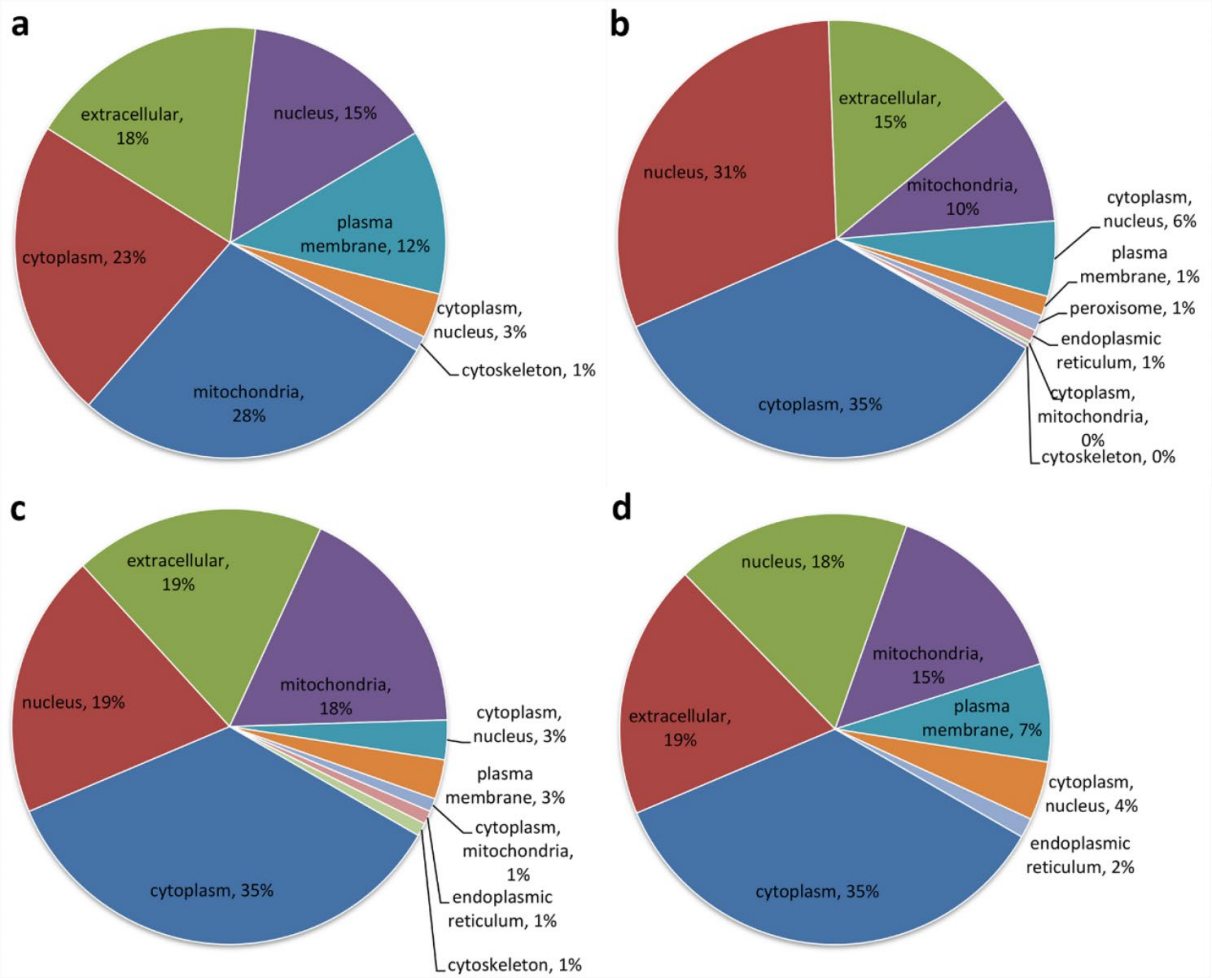
Results of subcellular location classification of down-regulated proteins in semi-buoyant and adhesive eggs of *C. alburnus* at three time stages of unfertilized (Un-FE), and 0, and 30 min post fertilization (0 min-FE and 30 min-FE). a and c, down-regulated proteins at Un-FE relative to 0 min-FE in semi-buoyant and adhesive eggs, respectively; b and d, down-regulated proteins at 0 min in comparison with 30 min-FE in semi-buoyant and adhesive eggs, respectively

In the classification of subcellular locations, the DRPs of unfertilized eggs relative to eggs at 0 min-FE were primarily located in mitochondria (28%), cytoplasm (23%), and extracellular (18%) in semi-buoyant eggs (Fig. 3a), and in cytoplasm (35%), nucleus (19%), and extracellular (19%) in adhesive eggs (Fig. 3c). The DRPs of eggs at 0 min-FE in comparison with 30 min-FE were mainly located in cytoplasm (35%), nucleus (31%), and extracellular (15%) in semi-buoyant eggs (Fig. 3b), and in cytoplasm (35%), extracellular (19%), and nucleus (18%) in adhesive eggs (Fig. 3d).

### Analyses of functional enrichment of down-regulated proteins

**Fig. 4:**
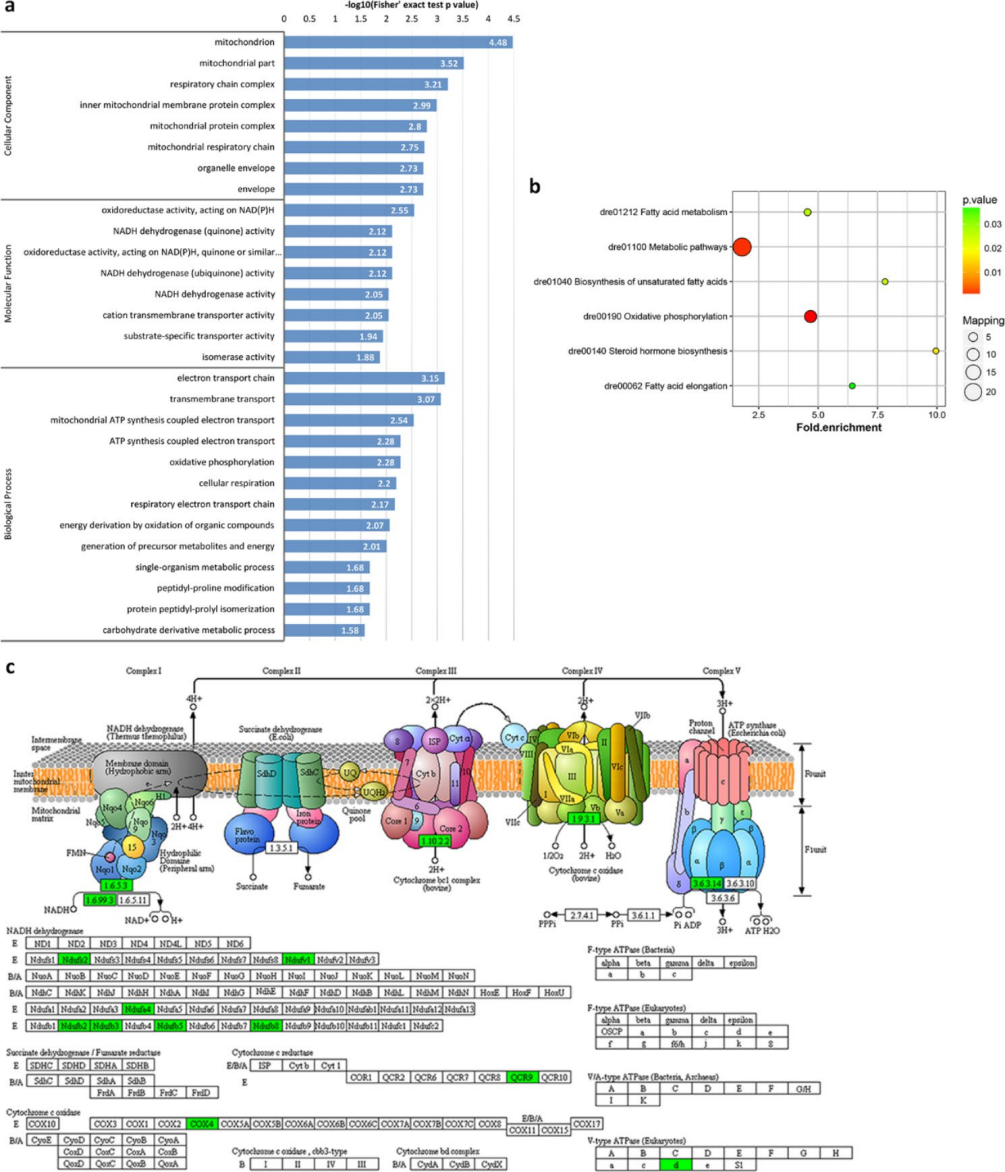
Results of functional enrichment of down-regulated proteins in unfertilized semi-buoyant eggs of *C. alburnus* relative to 0 min post fertilization. a, GO enrichment; b, KEGG pathway enrichment; c, oxidative phosphorylation pathway, modified from KEGG map00190. Green boxes indicate down-regulated proteins

**Fig. 5:**
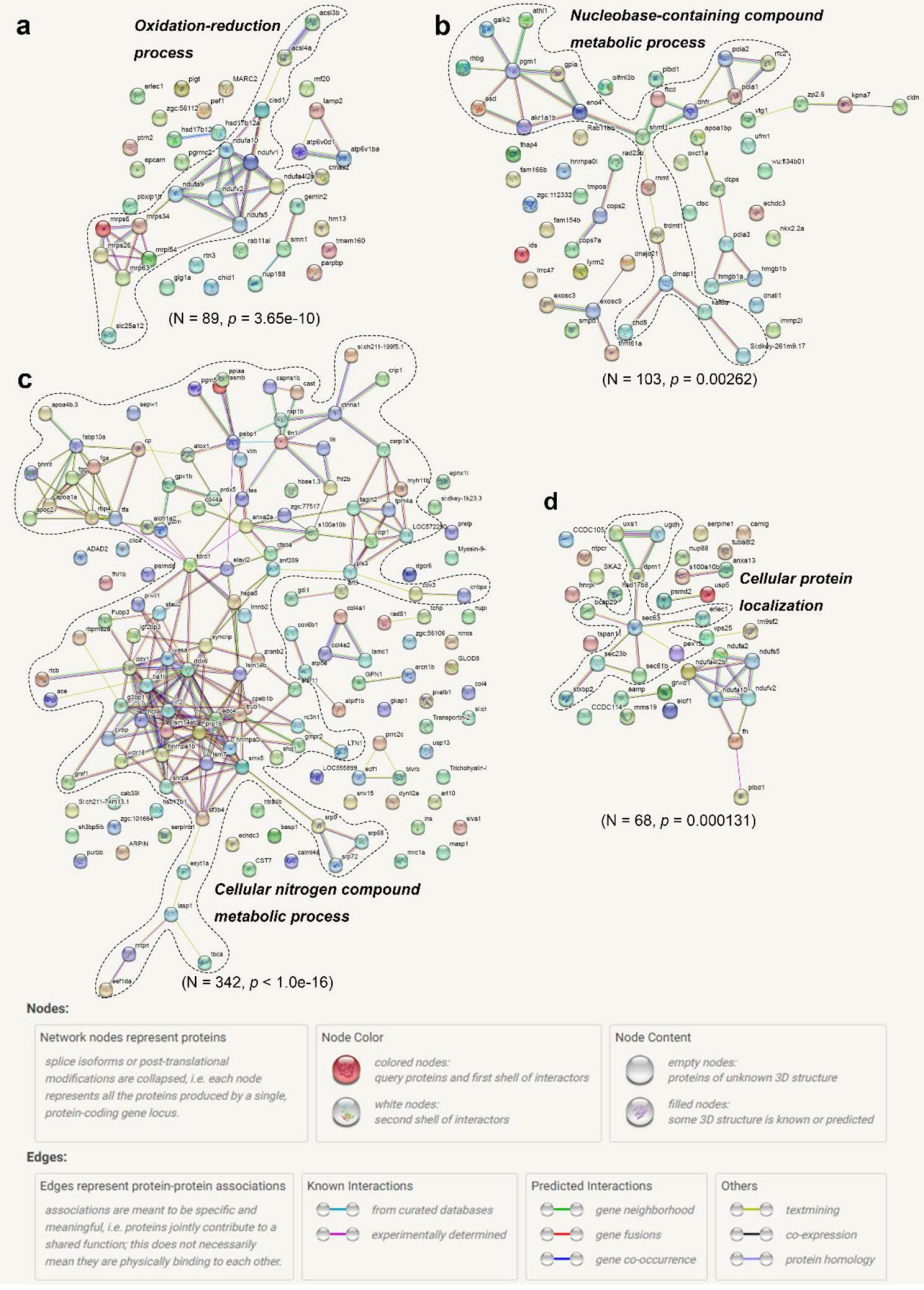
Protein-protein interaction network analysis of down-regulated proteins. a, PE-Un-FE vs. PE-0 min-FE; b, AE-Un-FE vs. AE-0 min-FE; c, PE-0 min-FE vs. PE-30 min-FE; d, AE-0 min-FE vs. AE-30 min-FE. Dashed lines encompass clusters of interacting proteins involved in physiological processes. Network nodes (spheres) represent all the proteins produced by a single, protein-coding gene locus, excluding splice isoforms or post-translational modifications. Each node is named for the zebrafish proteins to which spectra were mapped (see Additional file 1 for full protein names). Edges (colored lines) represent protein-protein associations which are meant to be specific and meaningful. Model statistics are presented at the bottom of each figure

**Fig. 6:**
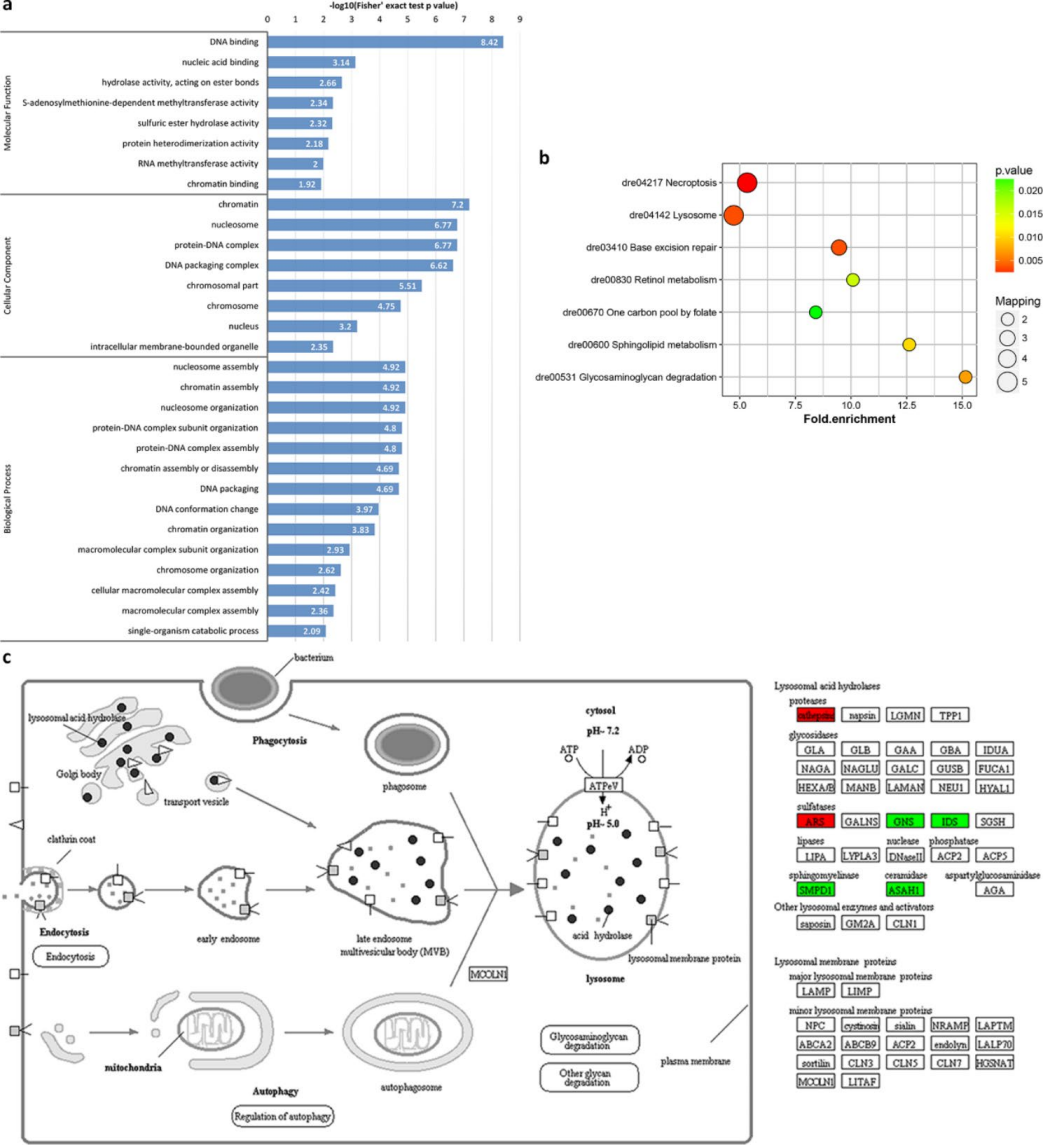
Results of functional enrichment of down-regulated proteins in unfertilized adhesive eggs of *C. alburnus* relative to 0 min post fertilization. a, GO enrichment; b, KEGG pathway enrichment; c, lysosome pathway, modified from KEGG map04142. Green boxes indicate down-regulated proteins; red boxes indicate up-regulated proteins

In a comparison of unfertilized eggs with eggs at 0 min-FE, the analysis of gene ontology (GO) enrichment showed that the DRPs of semi-buoyant eggs were more abundant in mitochondrion and respiratory chain complexes at the cellular level, oxidoreductase activity and NADH dehydrogenase activity at the molecular functional level, and electron transport chain, transmembrane transport and mitochondrial ATP synthesis coupled electron transport at the biological process level (Fig. 4a). Based on the Kyoto Encyclopedia of Genes and Genomes (KEGG) pathway enrichment, the DRPs of semi-buoyant egg changes were primarily mapped to the oxidative phosphorylation and fatty acid metabolism pathways (Fig. 4b). The proteins of NADH dehydrogenase (ubiquinone) Fe-S protein 2 (Ndufs2), NADH dehydrogenase (ubiquinone) flavoprotein 1 (Ndufv1), NADH dehydrogenase (ubiquinone) 1 alpha subcomplex subunit 4 (Ndufa4), NADH dehydrogenase (ubiquinone) 1 beta subcomplex subunit 2/3/5/8 (Ndufb2/3/5/8), ubiquinol-cytochrome c reductase subunit 9 (QCR9), cytochrome c oxidase subunit 4 (COX4), and V-type H^+^-transporting ATPase subunit d (V-ATPase d) were significantly down-regulated in the oxidative phosphorylation pathway (Fig. 4c). The protein-protein interaction (PPI) network analysis showed that the cluster in the network included proteins mainly related to the oxidation-reduction process. (Fig. 5a). At the same time, GO enrichment showed that the DRPs of adhesive eggs were mostly enriched in DNA and nucleic acid bindings, hydrolase activity at the molecular function level, chromatin, nucleosome and protein-DNA complex at the cellular component level, and nucleosome and chromatin assembly and nucleosome organization at the biological process level (Fig. 6a). KEGG pathway enrichment showed that the DRPs of adhesive eggs were mostly mapped to the necroptosis and lysosome pathways (Fig. 6b). The proteins of cathepsins and arylsulfatase (ARS) were significantly up-regulated, while the proteins N-acetylglucosamine-6-sulfatase (GNS), iduronate 2-sulfatase (IDS), sphingomyelin phosphodiesterase 1 (SMPD1) and acid ceramidase 1 (ASAH1) were down-regulated in the lysosome pathway (Fig. 6c). The PPI network analysis showed that the cluster in the network included proteins whose major functions are mostly related to the nucleobase-containing compound metabolic process(Fig. 5b).

**Fig. 7:**
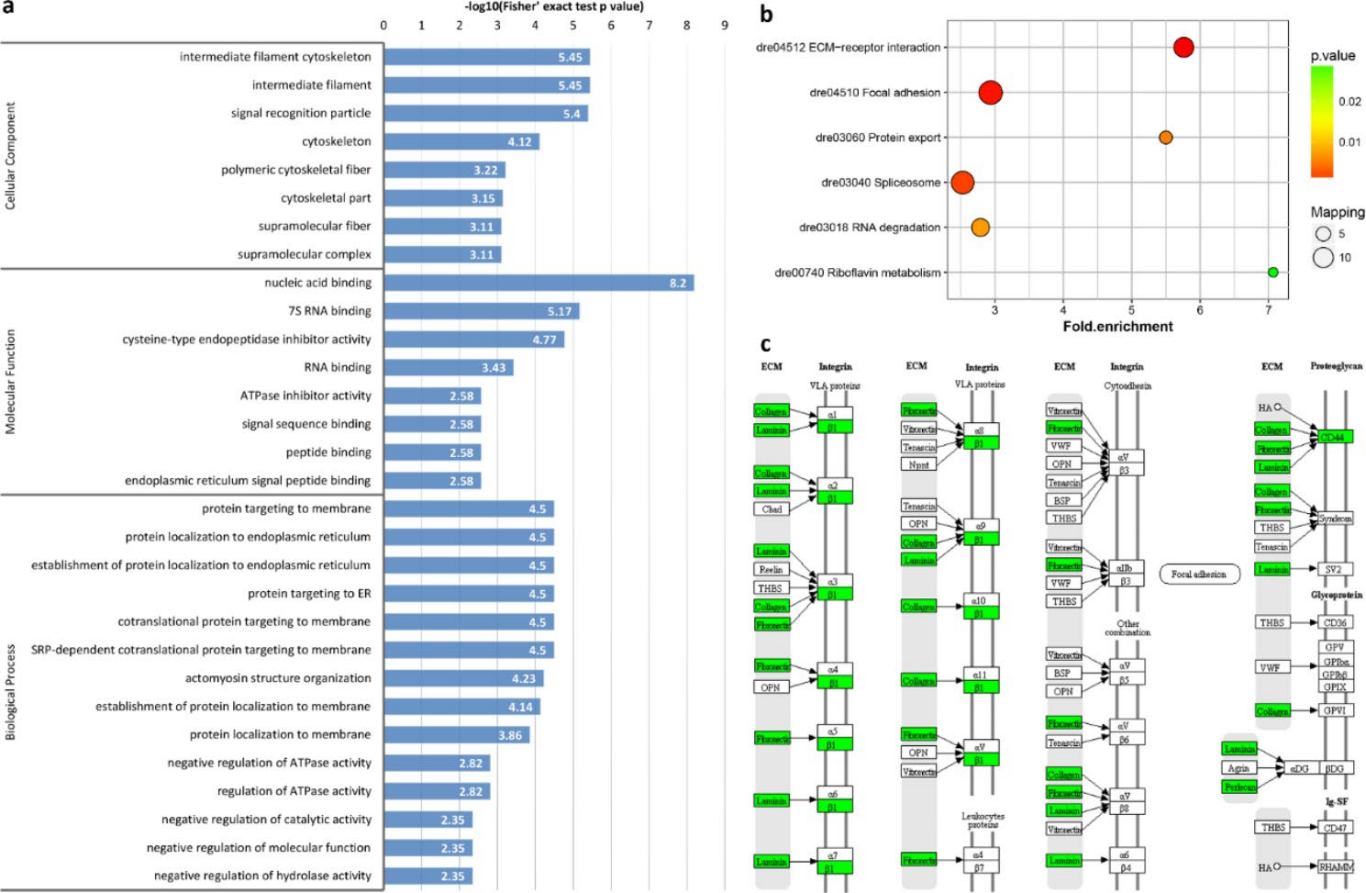
Results of functional enrichment of down-regulated proteins in semi-buoyant eggs of *C. alburnus* at 0 min post fertilization in comparison with 30 min post fertilization. a, GO enrichment; b, KEGG pathway enrichment; c, ECM-receptor interaction pathway, modified from KEGG map04512. Green boxes indicate down-regulated proteins

**Fig. 8:**
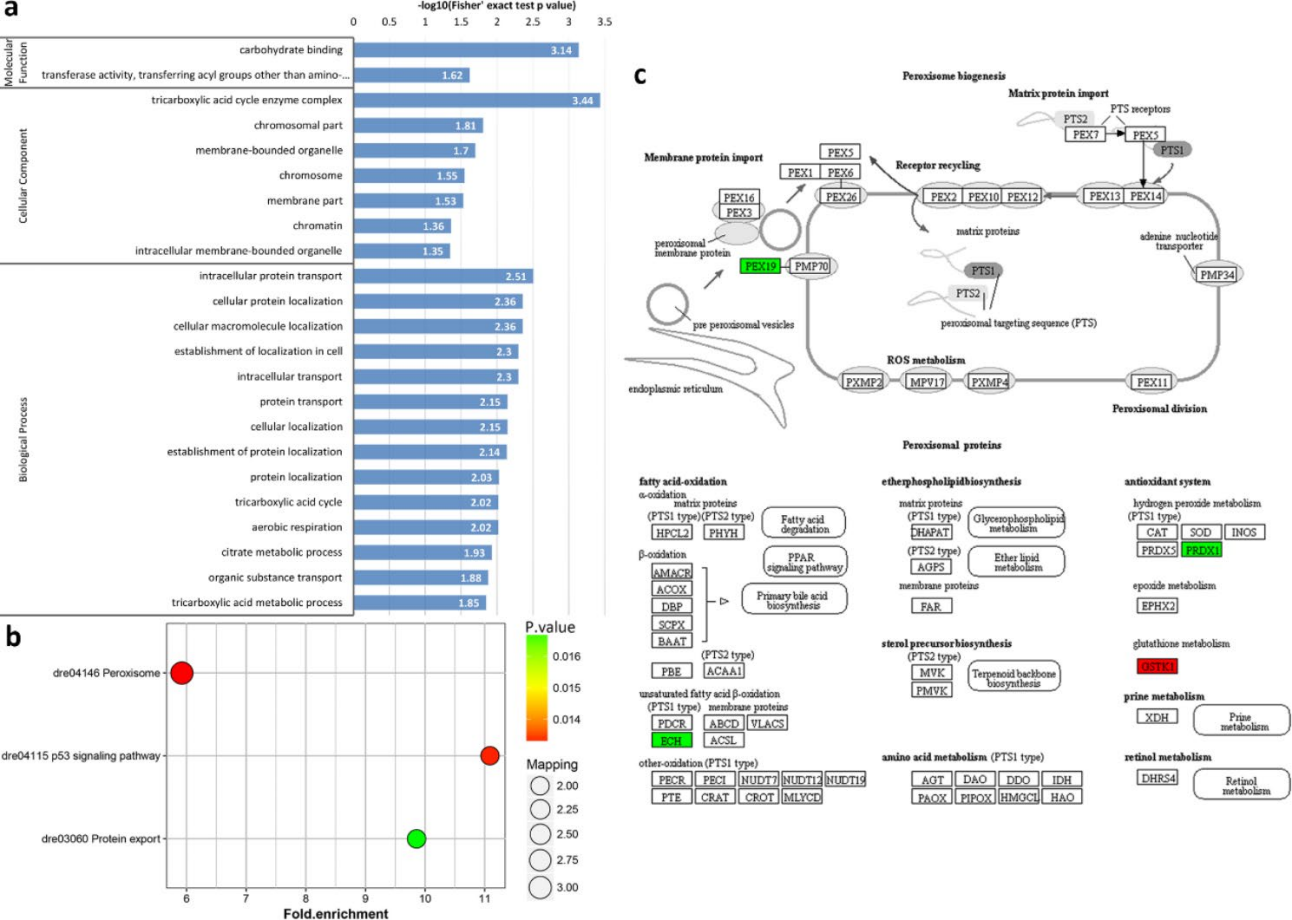
Results of functional enrichment of down-regulated proteins in adhesive eggs of *C. alburnus* at 0 min post fertilization in comparison with 30 min post fertilization. a, GO enrichment; b, KEGG pathway enrichment; c, peroxisome pathway, modified from KEGG map04146. Green boxes indicate down-regulated proteins; red boxes indicate up-regulated proteins

Regarding differences between 0 and 30 min-FE, GO enrichment showed that the DRPs of semi-buoyant eggs were more abundant in intermediate filament and cytoskeleton at the cellular component level, and protein targeting to membrane and protein localization to endoplasmic reticulum at the biological process level (Fig. 7a). Based on KEGG pathway enrichment, the DRPs of semi-buoyant eggs changed in the ECM-receptor interaction and protein export pathways, and mostly mapped to the focal adhesion pathway (Fig. 7b). The proteins of Collagen α1/α2/β1, Laminin α5/β1/γ1, Fibronectin, Perlecan, Integrin β1 and CD44 were significantly down-regulated in the ECM-receptor interaction pathway (Fig. 7c). The cluster in the PPI network included proteins that were mainly involved in the cellular nitrogen compound metabolic process (Fig. 5c). In contrast, the analysis of GO enrichment showed that the DRPs of adhesive eggs were mostly enriched in carbohydrate binding and transferase activity at the molecular function level, tricarboxylic acid cycle enzyme complex and membrane-bounded organelle at the cellular component level, and intracellular protein transport, cellular protein localization, cellular macromolecule localization and tricarboxylic acid cycle at the biological process level (Fig. 8a). Changes associated with the DRPs of adhesive eggs were primarily mapped to the peroxisome and protein export pathways based on KEGG pathway enrichment (Fig. 8b). The protein glutathione S-transferase kappa 1 (GSTK1) was significantly up-regulated, while the proteins peroxin-19 (PEX19), Delta3,5-Delta2,4-dienoyl-CoA isomerase (ECH) and peroxiredoxin 1 (PRDX1) were down-regulated in the peroxisome pathway (Fig. 8c). In the PPI network analysis, the cluster in the network included proteins mainly related to the cellular protein localization (Fig. 5d).

## Discussion

Although the processes of fertilization and egg activation are recognized as fundamental developmental events that are vital for early embryogenesis, the signal pathways of these processes in economically important fish species are poorly understood. Different modes of spawning in fish have evolved as adaptions to a variety of aquatic habitats. Semi-buoyant eggs represent an adaptation to fast-flowing rivers, while adhesive eggs are associated with lentic environments [9, 24, 26]. The signal pathways underlying fertilization and egg activation of different spawning modes have hitherto been overlooked. In this study, we investigated phenotypic traits, ultrastructure and protein expression levels of semi-buoyant and adhesive eggs of *C. alburnus*, comparing unfertilized eggs, and eggs at 0 and 30 min-FE.

Our results showed that the DRPs of semi-buoyant eggs of *C. alburnus*, at the stages of Un-FE relative to 0 min-FE, were primarily located in mitochondria and cytoplasm, and enriched in the oxidative phosphorylation and fatty acid metabolism pathways. Based on transcriptome analyses, differentially expressed transcripts were also enriched in the oxidative phosphorylation and fatty acid metabolism pathways during fertilization in the eggs of rainbow trout (*Oncorhynchus mykiss*) [33] and Atlantic cod (*Gadus morhua*) [34]. Mitochondria serve in supplying energy to the cell, producing cellular ATP through the highly efficient chemiosmotic coupling of electron and proton transfer to ATP synthesis on the inner mitochondrial membrane [35]. The translocation of protons from the mitochondrial matrix to the mitochondrial intermembrane space is mostly driven by multi-subunit oxidative phosphorylation protein complexes, which generate ATP from ADP [36]. Pyridine nucleotides (NADH) and flavins (FADH_2_) produced from fatty acid metabolism supply electrons to the electron transport system for oxidative phosphorylation and ATP synthesis [37]. These physio-biochemical processes involved in the oxidation-reduction were identified in the PPI network analysis. Our findings indicate that semi-buoyant eggs of *C. alburnus* activated energy metabolism at fertilization, possibly to facilitate the hatching process, which typically occurs more rapidly in lotic habitats and in eggs suspended in the water column because of more rapid development owing to greater oxygen availability [9, 38]. Notably, observations from SEM showed large pore openings at high density at the interior of the vestibule in adhesive eggs of *C. alburnus*. These large pore openings probably facilitate provision of oxygen to adhesive eggs in a lentic environment in which oxygen may be limiting. This conformation contrasts with more sparse pores in semi-buoyant eggs, which receive better oxygenation through drifting in the water column.

In adhesive eggs of *C. alburnus*, the DRPs of Un-FE compared with 0 min-FE were primarily located in the cytoplasm and nucleus, mostly enriched with DNA and nucleic acid bindings, and nucleosome and chromatin assembly, and mainly clustered in the nucleobase-containing compound metabolic process, which probably reflects the process of sperm-egg fusion during fertilization [6, 39, 40]. Sperm-egg fusion begins between the microvilli of the sperm entry site and the sperm plasma membrane [41], and triggers the initiation of the transition from meiosis to mitosis [42]. As maternal and paternal nucleosome and chromatin are extensively modified following fertilization, the genome is reprogrammed for totipotency. The DRPs of adhesive eggs of *C. alburnus* at the Un-FE stage relative to 0 min-FE were mostly mapped to the lysosome pathway, and enriched in hydrolase activity, suggesting material originating from the cortical reaction probably block the micropylar canal to prevent polyspermy [6]. Similar physiological changes were detected in monospermic teleost fishes and amphibians [40, 43]. Based on the ultrastructure of semi-buoyant and adhesive eggs, we showed that both rapidly formed a fertilization plug from the cortical vesicle after fertilization to block polyspermy [24]. The cortical vesicle contents released from the egg contain sperm-agglutinating chemicals that cause sperm to lose motility [44]. The block to polyspermy is a critical process for monospermic fertilization in most teleost fishes. Examination of ultrastructure also showed that the vestibule of adhesive eggs was recessed and wider than that of semi-buoyant eggs, which appears adaptive in adhesive eggs in facilitating sperm entry to attached eggs, in contrast with semi-buoyant eggs in which mixing of eggs and sperm during mating likely gives greater assurance of fertilization. However, the guidance mechanisms for sperm entry during fertilization in fishes remains relatively poorly understood [45, 46], and the adaptive significance, if any, of the contrasting forms of vestibule between adhesive and semi-buoyant eggs of *C. alburnus* remains to be demonstrated.

After fertilization, our results showed that both semi-buoyant and adhesive eggs of *C. alburnus* underwent rapid hydration with the formation of a perivitelline space. The perivitelline space may play an important role in protection, nutrition, flotation and preventing polyspermy during fish egg development [47]. The egg diameter of semi-buoyant eggs increased substantially after fertilization. A large perivitelline space is adaptive for these eggs by reducing their density and thereby enabling them to achieve partial buoyancy in a lotic habitat [48]. Based on the analysis of functional enrichment, the DRPs of semi-buoyant eggs at 0 min-FE in comparison with 30 min-FE were mostly enriched in intermediate filament, which comprise multiple strands of fibrous proteins, and cytoskeleton, with a significant change in the pathways of ECM-receptor interaction and focal adhesion. This result corresponds with findings using transcriptomic analysis during oocyte maturation and hydration in *Epinephelus coioides* [49] and *Gadus morhua* [50]. The proteins of intermediate filament and cytoskeleton probably play a role in maintaining the shape of fish eggs [6], which may be critical for semi-buoyant eggs that swell substantially to achieve buoyancy. The focal adhesion and ECM-receptor interaction pathways are likely involved in the immune system of the egg to prevent bacterial infection [51], and stretching of the egg envelope during swelling. Thus, the proteins of semi-buoyant eggs of *C. alburnus* that are enriched in cytoskeleton and ECM-receptor interaction pathways may function primarily to facilitate the rapid development of a large perivitelline space.

Proteomics analysis showed that the DRPs of adhesive eggs at 0 min-FE relative to 30 min-FE were mostly enriched in the tricarboxylic acid cycle and membrane-bounded organelle, and mapped mainly to the peroxisome pathway. Peroxisome is a small membrane-bounded organelle, which is present in virtually all eukaryotic cells and plays an essential role in lipid metabolism, especially fatty acid β-oxidation [52, 53]. The acetyl coenzyme A produced by fatty acid β-oxidation takes part in the tricarboxylic acid cycle through the glyoxylate cycle [54]. These findings suggest that adhesive eggs of *C. alburnus* possess elevated lipid metabolism, which is involved in the degradation of yolk lipids during early embryogenesis [55, 56]. Stereomicroscope results also showed that adhesive eggs of *C. alburnus* were opaque after fertilization, which further indicates that adhesive materials are excreted to the outside of the egg during the cortical reaction, covering the surface of the egg entirely [6, 57]. Based on proteomics analysis of functional enrichment, the DRPs of adhesive eggs at 0 min-FE relative to 30 min-FE were also mostly enriched in carbohydrate binding, cellular protein transport and localization, cellular macromolecule localization and protein export. Further, the PPI network analysis showed the cluster in the network that included proteins related to the cellular protein localization, suggesting these pathways may relate to the formation of adhesion and hardening in the egg envelope. Thus, during early embryogenesis adhesive eggs of *C. alburnus* appear to increase protein expression for lipid metabolism and egg envelope adhesion and hardening. These adaptations, which were not observed in semi-buoyant eggs to the same degree, imply that adhesion and hardening are more critical adaptations for eggs placed on a hard substrate, where they potentially risk damage from abrasion.

## Conclusion

The phenotypic traits, ultrastructure and protein expression levels of semi-buoyant and adhesive eggs of *C. alburnus* were investigated in unfertilized eggs, and eggs at 0 and 30 min-FE. Our study revealed that semi-buoyant eggs activated energy metabolism following fertilization and increased protein expression of the cytoskeleton and ECM-receptor interaction, which may facilitate egg swelling to achieve partial buoyancy in a lotic habitat. In contrast, adhesive eggs initiated the process of sperm-egg fusion and blocking of polyspermy following fertilization, and showed enhanced protein expression of lipid metabolism and formation of egg envelope adhesion and hardening, which likely facilitate development in reduced oxygen conditions and on a hard substrate. Although the pathways of these physiological events need further investigation, particularly regarding their functionality, our results are the first to highlight alternative signal pathways that show contrasting patterns during the processes of fertilization and egg activation for the different spawning modes of *C. alburnus*. Consequently, these results provide a novel insight into the adaptive significance of alternative pathways in the early embryogenesis of teleost fish, which contributes to our current understanding of fish reproduction.

## Methods

### Sample collection

Eggs of *C. alburnus* were collected from fish culture facilities in Jinzhou and Ezhou, Hubei Province, from May to July. Mature females and males were injected with the maturation-inducing steroid (MIS) 17α, 20β-dihydroxy-4-pregnen-3-one (330 µg kg^− 1^ body weight) to stimulate spawning. Approximately 12 h later, unfertilized eggs were expressed by gently pressing the female’s abdomen. Ovulated eggs were fertilized and collected at 0 and 30 min post fertilization. One portion of the samples was measured immediately for egg diameter and fixed in 2.5% glutaraldehyde at 4 °C, and the remaining eggs were immediately frozen in liquid nitrogen and stored at -80 °C until analysis. Absolute fecundity of mature females varied from 80,790 to 246,360, and relative fecundity ranged from 46.58 to 108.26 per gram in weight. First cleavage of embryo began at 30 min post fertilization at 26–27 °C, and hatching time was approximately 22 h [58].

### Stereomicroscope observation and egg diameter measurement

Photographs of eggs of *C. alburnus* were obtained with a stereomicroscope (MODEL C-BD230, Nikon, Japan) at the unfertilized stage, and 0 and 30 min post fertilization. Egg diameter was measured using the ImageJ analysis software (k 1.45, National Institutes of Health, USA).

### Scanning electron microscopy

For SEM, the three groups of fish eggs were fixed in 2.5% glutaraldehyde solution at 4 °C for 24 h, and then fixed in 1% osmium tetroxide in 0.1 M phosphate buffer (pH = 7.4). The specimens were dehydrated in a graded ethanol series, transferred from absolute ethanol into amyl acetate, dried under CO_2_, mounted on stubs, and metallized with gold. The prepared samples were observed using a HITACHI SU-8010 scanning electron microscope.

### TMT-based quantitative proteomic analysis

#### Protein extraction and measurement

TMT-based quantitative proteomics was performed to examine differences in the expression of proteins in semi-buoyant and adhesive eggs of *C. alburnus* at three time points (unfertilized, 0 and 30 min post fertilization). Samples in four volumes of lysis buffer (8 M urea, 1% Protease Inhibitor Cocktail) placed on ice were sonicated three times using a high-intensity ultrasonic processor (Scientz). The remaining debris was removed by centrifugation at 12,000 g at 4 °C for 10 min. Finally, the supernatant was collected, and the protein concentration of the eggs was determined with a BCA kit following the manufacturer’s instructions.

#### Trypsin digestion

The protein solution was reduced with 5 mM dithiothreitol for 30 min at 56 °C and alkylated with 11 mM iodoacetamide for 15 min at room temperature in darkness. The protein sample was then diluted by adding 100 mM triethylamonium bicarbonat (TEAB) to a urea concentration less than 2 M. Trypsin was added at 1:50 trypsin-to-protein mass ratio for the first digestion overnight at 37 °C and 1:100 trypsin-to-protein mass ratio for a second 4 h -digestion.

#### TMT labeling and HPLC fractionation

After trypsin digestion, the peptide mix was desalted by Strata X C18 SPE column (Phenomenex) and vacuum-dried. The peptide mix was reconstituted in 0.5 M TEAB, and labeled according to the manufacturer’s protocol for the TMT kit. Briefly, one unit of TMT reagent was thawed and reconstituted in acetonitrile. The peptide mixtures were then incubated for 2 h at room temperature and pooled, desalted and dried by vacuum centrifugation.

Based on high pH reverse-phase HPLC (High performance liquid chromatography), the tryptic peptide mixtures were fractionated using Agilent 300Extend C18 column (5 μm particles, 4.6 mm ID, 250 mm length). Peptide mixtures were first separated into 60 fractions with a gradient of 8–32% acetonitrile (pH = 9.0) over 60 min and combined into 24 fractions and dried by vacuum centrifuging.

#### LC-MS/MS analysis and database search

The tryptic peptide mixtures were dissolved in 0.1% formic acid, and separated using an EASY-nLC 1000 UPLC system. Solvent A contains 0.1% formic acid and 2% acetonitrile. The gradient comprised an increase from 9 to 25% solvent B (0.1% formic acid in 98% acetonitrile) over 26 min, 25–38% in 8 min, climbing to 80% in 3 min after which it was held at 80% for the last 3 min, all at a constant flow rate of 700 nL/min. The peptides were subjected to an NSI source followed by tandem mass spectrometry (MS/MS) in Q Exactive™ Plus (Thermo) coupled online to the UPLC. The electrospray voltage applied was 2.0 kV. The m/z scan range was 350 to 1550 for a full scan, and intact peptides were detected in the Orbitrap at a resolution of 60,000. Peptides were selected for MS/MS using an NCE setting of 100 and the fragments were detected in the Orbitrap at a resolution of 30,000. A data-dependent procedure that alternated between one MS scan followed by 20 MS/MS scans with 15.0 s dynamic exclusion. Automatic gain control was set at 5E4. Fixed first mass was set as 100 m/z.

The resulting MS/MS data were processed using the Maxquant search engine (v.1.5.2.8). Tandem mass spectra were searched against *C. alburnus* in the NCBI database (PRJNA819171, 189,877 sequences) concatenated with reverse decoy database. Trypsin/P was specified as a cleavage enzyme allowing up to 2 missing cleavages. The mass tolerance for precursor ions was set as 20 ppm in the First search and 5 ppm in the Main search, and the mass tolerance for fragment ions was set as 0.02 Da. Carbamidomethyl on Cys was specified as fixed modification, and oxidation on Met was specified as variable modification. False discovery rate (FDR) of protein and peptide/spectrum match (PSM) identification was adjusted to < 1%. Proteins in fish eggs with a fold change > 1.2 or < 0.83 and *p* < 0.05 were considered differentially expressed.

#### Protein annotation and functional enrichment analysis

The functions of differentially expressed proteins were determined via GO and KEGG pathway analyses and functional enrichment analysis. The GO classification was performed using the UniProt-GOA database (http://www.ebi.ac.uk/GOA/) and InterProScan software based on three categories: biological process, cellular component and molecular function. The KEGG classification was performed using the KEGG online service tools, KAAS, to annotate protein descriptions from the KEGG database, and then mapping the annotation result on the KEGG pathway database using the KEGG online service tool KEGG mapper. Wolfpsort (PSORT/PSORT II) was used to predict subcellular localization of proteins. For quality control purposes in egg proteomics, the mass error was close to zero and mostly less than 10 ppm, and the lengths of most peptides were distributed between 8 and 20 amino acid residues, indicating that the results met the standard.

#### Protein-protein interaction networks analysis

All down-regulated proteins among unfertilized eggs, and eggs at 0 and 30 min post fertilization were additionally used for the protein-protein interaction network analysis using STRING (v.11.5), a free biological database and web source (https://cn.string-db.org/) [59]. The data setting Confidence: Medium (0.40); Max Number of Interactions to Show: None/query proteins only. For the STRING analysis, only statistically significant enrichment results (*p* < 0.05) were represented.

### Statistical analyses

Statistical analyses of the data were performed using SPSS package 25.0 (SPSS, Chicago, IL, USA). All values are presented as the mean and standard deviation (SD). Normality and homogeneity of variances in the data were assessed with Kolmogorov-Smirnov and Levene’s tests, respectively. Significant quantitative differences between unfertilized, 0 and 30 min post fertilization groups of semi-buoyant and adhesive eggs of *C. alburnus* were identified using *t*-tests. For egg diameter, one-way analysis of variance (ANOVA) with Tukey’s multiple comparison test was employed to determine significant differences between results for the unfertilized and fertilized groups. Significant differences were set at *p* < 0.05 (*) and *p* < 0.01 (**).

## Electronic supplementary material

Below is the link to the electronic supplementary material.

Supplementary Material 1

## Data Availability

Raw peptide data are deposited at PRIDE (accession number: PXD023609). All data used and/or analyzed during the current study are available from the corresponding author on reasonable request.

## Abbreviations

ECM: Extracellular matrix
Un-FE: Unfertilized
0 min-FE: 0 min post fertilization
30 min-FE: 30 min post fertilization
TMT: Tandem mass tag
SEM: Scanning electron microscopy
URPs: Up-regulated proteins
DRPs: Down-regulated proteins
GO: Gene ontology
KEGG: Kyoto Encyclopedia of Genes and Genomes
Ndufs2: NADH dehydrogenase (ubiquinone) Fe-S protein 2
Ndufv1: NADH dehydrogenase (ubiquinone) flavoprotein 1
Ndufa4: NADH dehydrogenase (ubiquinone) 1 alpha subcomplex subunit 4
Ndufb2/3/5/8: NADH dehydrogenase (ubiquinone) 1 beta subcomplex subunit 2/3/5/8
QCR9: Ubiquinol-cytochrome c reductase subunit 9
COX4: Cytochrome c oxidase subunit 4
V-ATPase d: V-type H^+^-transporting ATPase subunit d
PPI: Protein-protein interaction
ARS: Arylsulfatase
GNS: N-acetylglucosamine-6-sulfatase
IDS: Iduronate 2-sulfatase
SMPD1: Sphingomyelin phosphodiesterase 1
ASAH1: Acid ceramidase 1
GSTK1: Glutathione S-transferase kappa 1
PEX19: Peroxin-19
ECH: Delta3,5-Delta2,4-dienoyl-CoA isomerase
PRDX1: Peroxiredoxin 1
MIS: Maturation-inducing steroid
TEAB: Triethylamonium bicarbonate
HPLC: High performance liquid chromatography
MS/MS: Tandem mass spectrometry
FDR: False discovery rate
PSM: Peptide/Spectrum match
ANOVA: Analysis of variance
